# The IMBG Test for Evaluating the Pharmacodynamic Response to Immunosuppressive Therapy in Kidney Transplant Patients: Current Evidence and Future Applications

**DOI:** 10.3390/ijms24065201

**Published:** 2023-03-08

**Authors:** Julio Pascual, Marta Crespo, Jose Portoles, Carlos Jimenez, Alvaro Ortega-Carrion, Teresa Diez, Isabel Portero

**Affiliations:** 1 Nephrology Department, Hospital 12 de Octubre, 28041 Madrid, Spain; 2 Nephrology Department, Hospital del Mar, Institut Mar for Medical Research, 08003 Barcelona, Spain; 3 Nephrology Department, Hospital Puerta de Hierro Mahadahonda, Institute IDHIPHIM for Medical Research, 28222 Madrid, Spain; 4 Nephrology Department, Hospital La Paz, 28046 Madrid, Spain; 5 Biohope Scientific Solutions for Human Health, 28760 Madrid, Spain

**Keywords:** transplant rejection, immunosuppressive therapy, infection, cellular pharmacodynamics, immune cell assay

## Abstract

Immunosuppressive drugs are widely used to prevent rejection after kidney transplantation. However, the pharmacological response to a given immunosuppressant can vary markedly between individuals, with some showing poor treatment responses and/or experiencing serious side effects. There is an unmet need for diagnostic tools that allow clinicians to individually tailor immunosuppressive therapy to a patient’s immunological profile. The Immunobiogram (IMBG) is a novel blood-based in vitro diagnostic test that provides a pharmacodynamic readout of the immune response of individual patients to a range of immunosuppressants commonly used in kidney transplant recipients. Here, we discuss the current approaches used to measure the pharmacodynamic responses of individual patients to specific immunosuppressive drugs in vitro, which can then be correlated with patient’s clinical outcomes. We also describe the procedure of the IMBG assay, and summarize the results obtained using the IMBG in different kidney transplant populations. Finally, we outline future directions and other novel applications of the IMBG, both in kidney transplant patients and other autoimmune diseases.

## 1. Introduction

The greatest risk faced by kidney transplant (KT) patients is graft loss due to immune rejection. After kidney transplantation, recipients undergo maintenance immunosuppressive combination therapy to reduce the risk of graft rejection and promote the long-term survival of the transplanted organ [1]. However, the persistent immunosuppression associated with these drug regimens carries certain risks, including the development of opportunistic infections and cancer.

Immunosuppressive therapies are empirically titrated according to clinical guidelines based on parameters, including the patient’s rejection risk profile, time since transplant, and drug plasma levels [2,3]. Drugs and doses may be adjusted in case of kidney dysfunction, the presence of donor-specific anti-HLA antibodies (DSA), histologic evidence of rejection, malignancies, or infection. The optimization of immunosuppressive therapy is crucial to find a middle ground between insufficient immunosuppression (resulting in rejection) and excessive immunosuppression (resulting in infections, malignancies, and toxicity) [4].

The immunosuppressive drugs recommended by clinical practice guidelines [2,3] to prevent graft rejection during maintenance therapy in KT patients (mycophenolate, cyclosporine, tacrolimus, everolimus, sirolimus and azathioprine) inhibit, via distinct mechanisms of action, the activity/proliferation of circulating T-lymphocytes (T cells). These immune system cells play a key role in initiating and mediating the alloimmune responses that characterize both acute rejection and chronic graft damage [5,6]. While antibody-mediated rejection is mediated by B-lymphocytes (B cells) that differentiate into anti-donor-specific antibody (DSA)-producing plasma cells, evidence indicates that CD4^+^ T cells appear to be essential for naive and memory DSA responses after transplantation [7,8].

Currently, the monitoring of immunosuppressive therapy is based on clinical and analytical follow-up and the determination of plasma drug levels, which serve as an indirect indicator of the extent of immunosuppression in a patient but provide no information on the drug’s effect on T cells. Notably, episodes of graft rejection and adverse effects are commonly described in patients with adequate plasma levels of immunosuppressive drugs [9]. Furthermore, a large body of evidence highlights marked discrepancies between these pharmacokinetic parameters and the clinical efficacy and safety of immunosuppressants in individual patients [10,11].

Several outputs from T-cell functional assays, including the ELISPOT (which enables the counting of cytokine-secreting memory T cells) [12] and Immuknow^®^ (which measures ATP production from CD4^+^ T cells) [13] assays, are used as pharmacodynamic readouts of immunosuppressant efficacy. However, while these assays provide information on the overall immunosuppression status of the transplant patient, they do not allow for a prediction of the patient’s response to specific immunosuppressive drugs or provide information on patient sensitivity to a given drug [14]. Tests that provide pharmacodynamic data on the inhibitory action of each immunosuppressant on patient T cells could help guide clinicians in their search for an optimal, personalized regimen [15,16].

The gold standard for the diagnosis of graft rejection is anatomopathological confirmation of rejection signs in a graft biopsy. However, biopsy is an invasive and costly procedure that is not free from complications and generally performed only when there are clear signs of a clinical or analytical deterioration indicative of probable renal injury (biopsy by indication) [2,3,17]. Such warning signs may emerge during patient follow-up and can be objectified by measuring of renal function parameters (plasma creatinine, proteinuria, glomerular filtration rate) or levels of recipient antibodies against donor HLA antigens (de novo donor-specific antibodies; dnDSA). dnDSA determination is routinely performed during patient follow-up and is an accepted marker of the risk of antibody-mediated rejection (ABMR) [18,19].

A few biomarkers have been developed for a noninvasive diagnosis of acute rejection in renal transplantation based on molecular or on functional tests, and some have been marketed for use in clinical practice. These are based on the analysis of HLA donor-specific antibodies and non-HLA antibodies, donor cell-free DNA, mRNA markers in blood and urine, gene transcriptomes, multiparametric biomarker panels, and pathology-based biomarkers [20,21,22,23,24,25,26,27,28,29]. While these tests provide information on the transplant recipient’s rejection risk and may serve as indirect indicators in cases of insufficient immunosuppression, they provide no information on the patient’s pharmacological response to individual immunosuppressants. In summary, diagnostic tools to help clinicians tailor immunosuppression to individual patients remain a key unmet clinical need.

In the first section of this article, we provide an overview of the results obtained using methods that assessed (i) in vitro pharmacodynamic responses (i.e., activity/proliferation of immunologically activated immune cells) upon exposure to individual immunosuppressants and (ii) the association between these responses and clinical outcomes in kidney and liver transplant patients. In the second section, we describe the IMBG methodology and associated data analysis.

Finally, we summarize the results obtained thus far using the IMBG and outline future directions for this technology.

## 2. Current Approaches for In Vitro Determination of Pharmacological Response to Immunosuppressants

Several assays purport to measure a patient’s level of immunosuppression by assessing the effects of individual immunosuppressive drugs on specific targets (e.g., on enzyme activity or T-cell subsets) in vitro, thereby providing a pharmacodynamic readout of immunosuppressant efficacy. Here, we summarize key findings from studies that assessed the in vitro activation/proliferation of immunologically stimulated patient PBMCs or mixed lymphocyte cultures exposed to individual immunosuppressants, and the risk of graft rejection and/or side effects due to immunosuppression (cytomegalovirus or other opportunistic infections, nephrotoxicity) in renal or liver transplant patients treated with those drugs.

In their studies, Langhoff et al. investigated the relationship between graft survival and in vitro lymphocyte sensitivity to steroids. They determined the concentrations of methylprednisolone that suppressed by 50% the in vitro proliferation response to phytohaemagglutinin (ED50) in mixed-lymphocyte cultures (MLC) from kidney transplant patients receiving immunosuppressive treatment (azathioprine and steroids). They also found that 1-year graft survival was significantly higher in patients with methylprednisolone ED50 values below the median ED50 (i.e., greater sensitivity) than in those with values above the median ED50 (86% vs. 29%; *p* < 0.0002) [30]. This effect was also observed in transplant recipients treated with cyclosporine A and steroids, but failed to reach significance. In line with these observations, Langhoff and Ladefoged [31] reported that lymphocyte cultures from azathioprine- or cyclosporine-A-treated patients with a functioning graft were 20 and 12 times more sensitive to methylprednisolone, respectively, than those of patients with graft failure at 6 months. Together, these findings indicate that the steroid sensitivity of recipients strongly influences kidney graft survival.

Francis et al. [32] examined whether preoperative in vitro sensitivity of MLC to prednisolone, cyclosporine A, and antithymocyte globulin in kidney transplant recipients that were treated with prednisolone, azathioprine, and cyclosporine A correlated with transplantation outcomes. So-called resistant patients (in whom one or more immunosuppressants failed to inhibit MLC proliferation by 50%) had a higher rate of graft loss due to acute rejection and more episodes of acute rejection. The authors concluded that patients at high risk of acute kidney graft rejection could be identified using a pretransplant in vitro assay, which could facilitate the choice of immunosuppressant therapy.

In the 1990s, Hirano et al. [33] evaluated the ability of four glucocorticoids (hydrocortisone, prednisolone, methylprednisolone, and dexamethasone) to suppress the in vitro blastogenesis of mitogen-stimulated PBMCs from chronic renal failure (CRF) patients and healthy volunteers by comparing the concentrations of steroids that caused 50% inhibition of lymphocyte blastogenesis (IC50). A significantly larger proportion of CRF patients compared to healthy volunteers showed a decrease in lymphocyte response to prednisolone (26.2% vs. 4.1%; *p* < 0.01). Interestingly, after kidney transplantation, CRF patients who showed an impaired pretransplant lymphocyte response to prednisolone had a significantly higher incidence of acute graft rejection while receiving immunosuppressive treatment with prednisolone and cyclosporine A [33]. These observations were in line with previous findings [34] showing a higher incidence of acute rejection in patients who were less sensitive to the inhibitory effect of prednisolone, as determined in pretransplant lymphocytes, compared with sensitive patients (63.6% vs. 16%; *p* < 0.05). Together, these results suggest an association between a poor in vitro response to prednisolone and risk of rejection.

Using the same methodology, Takeuchi et al. examined the influence of PBMC sensitivity to cyclosporine A on clinical outcomes after renal transplantation. Recipients were classified into low-, standard-, and high-sensitivity groups based on cyclosporine A IC50 values. Importantly, compared with the high-sensitivity group, the low-sensitivity group showed a higher incidence of graft failure (*p* < 0.05) during the 4 years post-transplantation and a higher incidence of renal dysfunction, as measured by plasma creatinine values after 2 years of follow-up (*p* < 0.05) [35].

Interestingly, cellular pharmacodynamic responses were also used to identify patients with a high risk of rejection after glucocorticoid withdrawal [36]. Median IC50 values for cortisol and methylprednisolone were significantly higher in recipients with decreased renal function due to glucocorticoid withdrawal than in those who had not experienced glucocorticoid withdrawal symptoms (cortisol, *p* < 0.001; prednisolone, *p* < 0.003). The authors concluded that the glucocorticoid pharmacodynamics of patient lymphocytes may constitute a reliable biomarker for the identification of KT recipients who will experience safe glucocorticoid reduction/withdrawal.

In a set of studies using Hirano’s methodology and the lymphocyte immunosuppressant sensitivity test (LIST), Sugiyama et al. analyzed the association between the in vitro PBMC proliferation response to different immunosuppressants and various clinical outcomes. No association was found between cyclosporine A mean IC50 values and incidence of acute graft rejection or cytomegalovirus (CMV) infection in a small population of 19 KT patients [37]. In a sample of 15 KT patients, individual differences in pre- versus post-transplant sensitivity to mycophenolic acid were observed, but there was no association with clinical outcomes (rejection, CMV incidence) [38]. However, the same authors later reported differences in mycophenolic acid IC50 values 2 weeks after transplant that were associated with CMV incidence (but not acute rejection) in a group of 16 KT patients [39]. Lastly, LIST determination of pre-transplant sensitivity to tacrolimus in 17 KT patients predicted acute rejection (but not CMV infection) in the first 3 months of follow-up after transplant [40].

Kurata et al. used a carboxyfluorescein diacetate succinimidyl ester (CFSE)-based T-cell proliferation assay to explore the clinical relevance of cellular pharmacodynamic responses to cyclosporine A in renal transplantation. Patients with a high pretransplant sensitivity to cyclosporine A tended to experience viral reactivation after transplant [41]. Moreover, an analysis of post-transplant samples showed that viral reactivation was associated with a high sensitivity to cyclosporine A and acute T-cell-mediated rejection (ATMR), as well as de novo DSA titers >1000 MFI during follow-up, with low sensitivity to cyclosporine A. The same authors explored the relationship between IC50 and cyclosporine-induced nephrotoxicity clinically and morphologically diagnosed in protocol biopsies performed 6 months and 1 year after transplantation and in indicated biopsies. They observed a significant association between cyclosporine-induced nephrotoxicity and lower IC50 (i.e., increased sensitivity to cyclosporine A) (*p* < 0.05) [42].

A crucial aspect to bear in mind that was not addressed in any of the aforementioned studies is the confounding effect that concomitant treatments traditionally prescribed for KT patients can have on clinical outcomes. Using the same methodology as Hirano’s group, a study of liver transplant patients treated with tacrolimus monotherapy measured pretransplant sensitivity to tacrolimus [43]. During the 4-week follow-up period after transplantation, patients with high in vitro sensitivity to tacrolimus had more opportunistic infections (*p* = 0.0401) while patients who were less sensitive experienced more graft rejections (*p* = 0.0297). Tacrolimus doses and plasma levels were similar in both groups.

In summary, despite small patient samples, short follow-up periods (often less than 1 year), and a low incidence of clinical events, these studies suggest an association between in vitro pharmacodynamic responses to individual immunosuppressants and clinical outcome variables after transplantation. Specifically, there is a greater risk of acute rejection and graft failure in patients with low sensitivity to the tested immunosuppressants and a greater risk of side effects in more sensitive patients (opportunistic infections and calcineurin-inhibitor-induced nephrotoxicity).

An important limitation to the clinical implementation of current pharmacodynamics-based approaches is that they do not enable simultaneous testing for several immunosuppressants. The IMBG test has been developed to overcome this limitation, since a single assay enables a comprehensive automated profiling of an individual patient’s sensitivity to a battery of immunosuppressive drugs, based on which clinicians can make better informed decisions regarding the patient’s current immunosuppressant regimen and/or potential alternative treatment options.

## 3. The Immunobiogram Test: Methodology and Data Analysis

**Figure 1:**
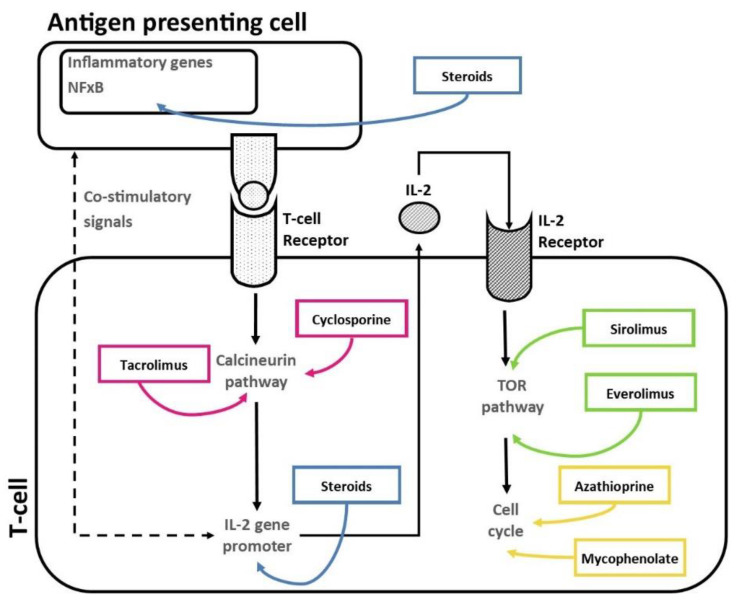
Mechanisms of action of immunosuppressive drugs on patient’s T cells.

The immunosuppressive drugs used to prevent graft rejection during maintenance therapy inhibit the activity/proliferation of circulating T cells via different mechanisms of action (Figure 1).

The IMBG is a functional in vitro assay that enables simultaneous quantitative measurement of a patient’s T cells response to a battery of immunosuppressive drugs commonly used in KT, currently including mycophenolate, tacrolimus, everolimus, sirolimus, and steroids [15,16]. The evaluation of PBMC responses is particularly relevant given that adaptive T cell response is a key initiator, mediator, and effector of alloimmune response and a key barrier to successful transplantation. Moreover, T lymphocytes are the target cells for the immunosuppressive drugs used in clinical practice to prevent kidney transplant rejection. Indeed, some of the pivotal drugs used in the IMBG assay exclusively target T-lymphocytes [6]. Likewise, T-lymphocytes are the predominant cell subset in PBMCs [44].

**Figure 2:**
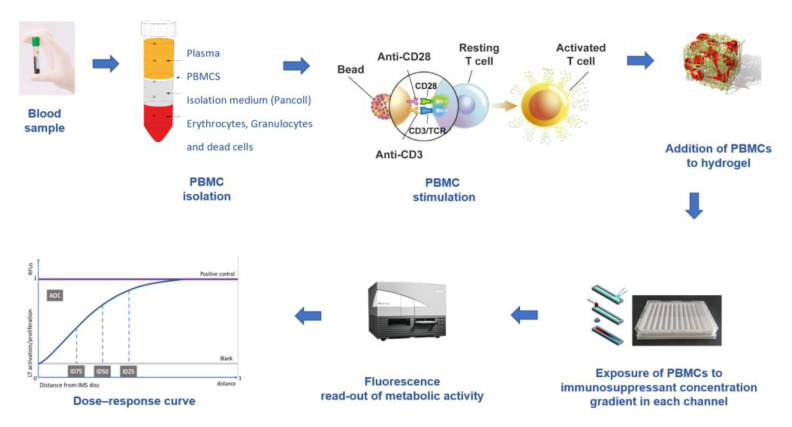
IMBG assay procedure. PBMCs are extracted from the patient’s blood sample and immunologically stimulated to induce their activation and proliferation. These activated PBMCs are embedded in a hydrogel substrate, which is then loaded into segregated channels in the IMBG plate. PBMCs in each channel are exposed to a concentration gradient of a distinct immunosuppressant. Next, PBMC activation and proliferation along the concentration gradient are measured using a resazurin-based immunofluorescence assay, which provides a read-out of PBMC response to each immunosuppressant. For each immunosuppressant, dose–response curves are generated based on 15 immunofluorescence readings taken at sequential points along the concentration gradient in the IMBG channel.

Because the IMBG is a novel test undergoing development, its experimental procedure has been modified since its inception to improve the analytical aspects of the technique. Here, we describe the IMBG methodology and data analysis as currently performed [16] (Figure 2).

**Figure 3:**
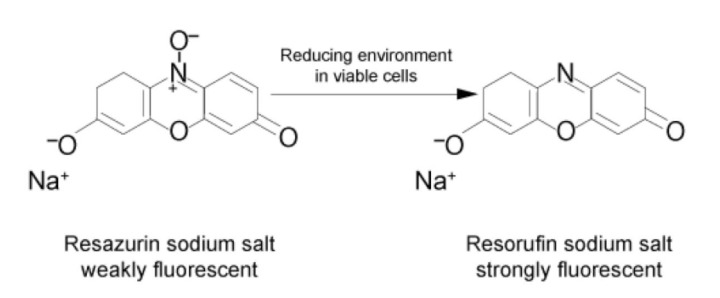
Measurement of metabolic activity. The measurement is performed by means of ubiquitous reducing agents in cells (NADH and NADPH) with of a vital probe (resazurin). Resazurin is reduced via the aerobic respiration of metabolically active cells, changing resazurin to resorufin, a reaction that fluoresces when exposed to green light.

Using a standard Ficoll™ gradient procedure, peripheral blood mononuclear cells (PBMCs) are isolated from the patient’s blood sample. PBMCs are then activated via incubation for 4 days in a standard incubator (37 °C, 5% CO_2_) in X-VIVO medium in the presence of agonistic antibodies (Dynabeads Human T-activator CD3/CD28) to induce their activation and proliferation. Activated PBMCs are embedded in a hydrogel substrate, which is added to segregated channels in the IMBG plate. Currently, the IMBG plate is designed to simultaneously test two control conditions (a positive control [C+], consisting of stimulated PBMCs, and a blank control, lacking PBMCs) and the following five immunosuppressant conditions: mycophenolic acid, tacrolimus, methyl prednisolone, sirolimus, and everolimus. Autoclaved paper discs used to deliver immunosuppressant drugs are placed at the end of the hydrogel+PBMC-loaded channels in the IMBG plate. No discs are placed in either of the two control channels (positive and blank controls). The passive diffusion of the immunosuppressant through the hydrogel generates a concentration gradient along which the activation/proliferation of the embedded PBMCs is inhibited in a dose-dependent manner. After placement of the discs loaded with the immunosuppressants of interest, the IMBG plate is incubated for approximately 15 h at 37 °C and 5% CO_2_. PBMC metabolic activity, which reflects activation and proliferation, is determined using a resazurin-based assay followed by measurement of PBMC fluorescence using a microplate reader [15] (Figure 3).

For each immunosuppressive drug, the IMBG acquires 15 sequential immunofluorescence readings along the concentration gradient in the IMBG channel, providing a read-out of PBMC activation/proliferation across the drug concentration gradient [15]. Fluorescence data are acquired and analyzed using proprietary software (IMBG Software Version: 3.0) and automatically normalized to a scale of 0–1 (1 = positive control value). A dose–response curve is generated by plotting the immunosuppressant concentration gradient (i.e., distance from immunosuppressant disc at which fluorescence reading is taken), normalized to a scale of 0–1 (0 and 1 equal points of maximum and minimum immunosuppressant concentration, respectively), against normalized fluorescence data.

Using the software, the following dose–response curve parameters are calculated in order to quantify the pharmacological response to each of the immunosuppressants tested [16]: (i) area over the curve (AOC—the degree of global PBMC inhibition in the presence of the immunosuppressant) and (ii) half-maximal (ID50), 25% maximal (ID25), and 75% maximal (ID75) inhibitory response (the points on the *X*-axis at which 50%, 25%, and 75% of PBMC are inhibited, respectively, are observed).

**Figure 4:**
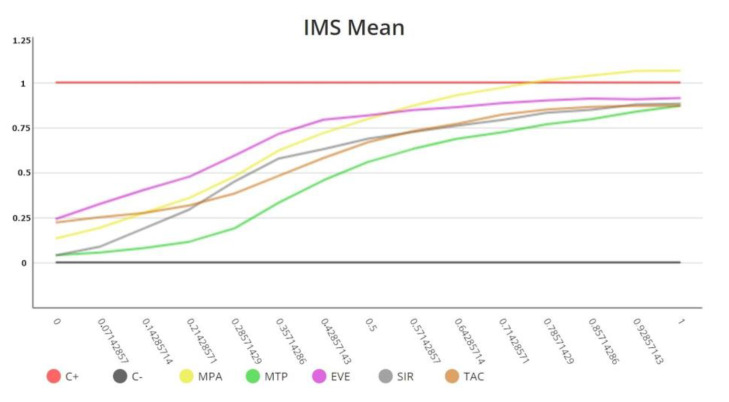
Immunobiogram dose–response curves obtained for a patient. Each immunosuppressive drug is tested in triplicate in the Immunobiogram plate, and the mean value of these measurements (IMS mean) is used to obtain the final curve for each immunosuppressant.

The AOC was used as the final classificatory variable owing to its discriminatory capacity and analytical stability (Figure 4).

Currently each immunosuppressive drug is tested by triplicate in the Immunobiogram plate, and the mean value of these measurements is used to obtain the final dose–response curve for each immunosuppressant. Triplicate curves hardly show any deviation and are always into the range approved by Regulatory Authorities in Europe and US, being Immunobiogram a robust functional assay.

## 4. The IMBG Measures In Vitro Sensitivity to Immunosuppressive Drugs in KT Patients

We previously used the IMBG to characterize the individual immunosuppressant response profile of 60 KT patients in a proof-of-concept study, demonstrating for the first time the assay’s clinical potential for the personalized management and monitoring of patients undergoing immunosuppressive therapy [15].

Recently, we reported the results of the larger scale TRANSBIO (BHP-IBG-2017-01) study, in which the IMBG was used to examine the association between patients’ sensitivity to their prescribed immunosuppressants and clinical outcomes (i.e., key clinical variables associated with graft rejection) [16]. This was an international, multicenter, observational study in a kidney transplant population undergoing maintenance immunosuppressive therapy. Patients were stratified by clinical course as follows: poor clinical course (PCC), i.e., patients with renal dysfunction, and rejection signs in biopsy or/and an increase in donor specific antibody (dnDSA) levels in last 12 months; good clinical course (GCC), i.e., patients with stable renal function and treatment, no previous rejection episodes, and no DSA titers. From the dose–response curves generated by the IMBG, four parameters were selected and compared between PCC and GCC patients treated with mycophenolate (*n* = 85), tacrolimus (*n* = 85), corticosteroids (*n* = 91), cyclosporine A (*n* = 14), or everolimus (*n* = 10). For all immunosuppressants and parameters, mean values were lower in the PCC (rejection) versus the GCC group, indicating a lower sensitivity to the prescribed medication in patients with PCC, though this difference was statistically significant only for specific parameters in patients treated with mycophenolate, tacrolimus, corticosteroids, cyclosporine A, and everolimus. Notably, univariate and multivariate logistic regression analyses confirmed independent associations between in vitro sensitivity (i.e., curve parameter values) and a clinical course that persisted after adjustment for concomitant treatments in the mycophenolate-, tacrolimus-, and corticosteroid-treated subgroups [16]. This observation is particularly relevant since conventional maintenance immunosuppressive therapy in KT patients involves combined treatment with different immunosuppressants; therefore, the potential confounding effects of co-administered drugs must be considered.

In the TRANSBIO study, the IMBG was assessed in a population of patients at either extreme of the clinical course spectrum [16]. Recently, we used the IMBG in a clinically representative sample of 210 KT patients in the maintenance phase (unpublished data). We observed a normal distribution of AOC values and the main IMBG curve parameter. Based on the mean and standard deviations of the AOC for each immunosuppressant tested, we calculated a Z-score that allowed us to position the patient response to each immunosuppressant relative to that of the reference population (renal transplant patients in the maintenance phase). This method expands upon the previously described applications of the IMBG and suggests that it could be incorporated into routine clinical practice as an automated method to quantitatively measure patient sensitivity to a panel of immunosuppressive drugs relative to reference population. This could provide valuable information based on which immunosuppression could be subsequently increased or decreased in patients at high- and low risk of rejection, respectively.

In summary, our observations thus far demonstrate that the IMBG can be used to quantify the pharmacodynamic response to individual immunosuppressive drugs and highlight its potential as a clinical tool for the personalized monitoring of kidney transplant patients receiving maintenance immunosuppression.

## 5. Future Directions

The improvement of outcomes in the field of transplantation depends on the development of new strategies to prevent graft loss and extend patient survival, combined with those that promote organ donation, maintenance, and distribution, and facilitate data analysis through optimized data collection in patient records [1].

The main challenge in monitoring transplant recipients is reducing the risk of graft rejection and minimizing complications related to excessive immunosuppression, ultimately extending graft and patient survival. The availability of additional information on the patient’s rejection risk profile and response to immunosuppressive therapy could help guide individualized adjustment of therapy and consequently improve clinical outcomes. The IMBG provides information on patient pharmacodynamic response to individual immunosuppressive drugs previously unavailable to clinicians.

Studies conducted using the IMBG assay have enabled the quantification of variations in the pharmacodynamic responses (i) of patients to distinct immunosuppressive drugs and (ii) of individual patients to their prescribed immunosuppressant. These findings also indicate that sensitivity to individual immunosuppressants in vitro, as measured by the IMBG, is correlated with clinical outcomes (rejection) in renal transplant recipients in the maintenance phase. Specifically, a lower sensitivity to the prescribed immunosuppressant is associated with a higher probability of rejection.

An observational, longitudinal, international study involving 16 reference centers, with a recruitment target of 450 patients, is currently underway, and plans to analyze IMBG tests performed before and 3, 6, 9 and 12 months after kidney transplantation. The goal of this study is to analyze IMBG data collected during the first year after transplantation and to assess the relationship between these data and rejection (biopsy-proven) or opportunistic infection during follow-up. Furthermore, response profiles will be modeled according to the patient’s baseline risk (of both rejection and adverse events due to immunosuppression), considering the pharmacokinetic and pharmacodynamic response to immunosuppressants and other markers commonly used in clinical practice (renal function, dnDSA, viral load, etc.). An extension of this study plans to obtain patient follow-up data for at least three years.

Other planned studies based on real-world evidence will analyze the long-term impact of the use of the IMBG in clinical practice, specifically its effect on the incidence and time until the occurrence of events, need for diagnostic tests, medication changes, direct and indirect costs, and patient quality of life. Existing pharmaco-economic models can quantify the impact of a decreased incidence of rejection episodes or adverse effects on medical care costs in kidney transplant recipients [45].

To date, the IMBG assay has not been used to analyze B-lymphocytes due to the low proportion of B cells relative to total lymphocytes in isolated PBMCS, as well as their greater instability in culture and in freezing conditions. While the inclusion of B-lymphocyte assays using other drugs is one possible future direction, the quantification of B cell responses would require a very different approach.

Future studies will assess the utility of the IMBG to guide the selection of combinations of immunosuppressant drugs based on patient sensitivity to each. Other goals include evaluating the use of IMBG in other types of transplant patients and in autoimmune pathologies other than renal diseases that are routinely treated with immunosuppressants, and in which an effective treatment response is critical to achieve faster remission and prevent recurrence.

## Data Availability

No new data were created or analyzed in this study. Data sharing is not applicable to this article.
